# Histone-acetylation: a link between Alzheimer's disease and post-traumatic stress disorder?

**DOI:** 10.3389/fnins.2014.00160

**Published:** 2014-06-24

**Authors:** Sanaz Bahari-Javan, Farahnaz Sananbenesi, Andre Fischer

**Affiliations:** ^1^Department of Psychiatry and Psychotherapy, University Medical Center GöttingenGöttingen, Germany; ^2^Research Group for Epigenetics in Neurodegenerative Diseases, German Center for Neurodegenerative Diseases (DZNE) GöttingenGermany

**Keywords:** Histone-deacetylases (HDACs), epigenetic gene-expression, HDAC inhibitors (HDACi), Alzheimer's disease (AD), post-traumatic stress disorder (PTST)

## Abstract

The orchestration of gene-expression programs is essential for cellular homeostasis. Epigenetic processes provide to the cell a key mechanism that allows the regulation of gene-expression networks in response to environmental stimuli. Recently epigenetic mechanisms such as histone-modifications have been implicated with cognitive function and altered epigenome plasticity has been linked to the pathogenesis of neurodegenerative and neuropsychiatric diseases. Thus, key regulators of epigenetic gene-expression have emerged as novel drug targets for brain diseases. Numerous recent review articles discuss in detail the current findings of epigenetic processes in brain diseases. The aim of this article is not to give yet another comprehensive overview of the field but to specifically address the question why the same epigenetic therapies that target histone-acetylation may be suitable to treat seemingly different diseases such as Alzheimer's disease and post-traumatic stress disorder.

## Introduction

The term epigenetics has been introduced by Conrad Waddington to describe heritable changes of a phenotype that does not depend on altered DNA-sequence (Holliday, 1994). The key epigenetics mechanisms are histone-modifications and DNA methylation. In addition to DNA-methylation it was recently discovered that DNA can be hydroxymethylated which is particularly prominent in brain tissue. Also the action of non-coding RNAs has been considered as an epigenetic process. Histone-modifications, DNA-methylation and non-coding RNAs act in concert to orchestrate gene-expression in the context of gene-environment interactions. Because complex neuropsychiatric diseases result from variable combination of genetic and environmental risk factors, epigenetic processes are gaining increasing interest in the neurosciences. Numerous review articles have addressed this issue (Abel and Zukin, 2008; Day and Sweatt, 2010; Fischer et al., 2010; Jakovcevski and Akbarian, 2012; Parkel et al., 2013; Rudenko and Tsai, 2014). Thus, the aim of this review is to give an overview specifically on the role of histone-modifications in neuropsychiatric diseases and ask the question why epigenetic therapies appear to be suitable for apparently different types of brain diseases, especially Alzheimer's disease (AD) and post-traumatic stress disorder (PTSD).

The DNA strand within a cell is tightly condensed which is achieved by compacting DNA into chromatin that consists of DNA bound to histone proteins. The 4 core histones (H) build the nucleosome around with 147 bp of DNA is wrapped. The histone tails are subjected to post-translational modification including acetylation, methylation, phosphorylated, ubiquitination, sumoylation, or ADP-ribosylation (Vaquero et al., 2003) which is mediated by the counteracting activities of enzymes that add or remove such modifications. As such, histone-acetylation is mediated by Histone-acetyltransferases (HAT) and Histone-deacetylases (HDACs). The activity of such enzymes affects the chromatin state and thus gene-expression. On one hand the modification of histone-tails alters the electrostatic interaction of DNA and the nucleosome and in addition the various combinations of histone-modifications give rise to a combinatorial pattern, the so called the “histone-code” (Strahl and Allis, 2000). Histone-acetylation and histone-methylation occurs predominantly at lysine residues (K). While only one acetyl group can be added to a given lysine within a histone tail, methylation can occur as mono-, di-, or tri-methylation. Specific histone-modifications have been linked to a particular mode of gene-expression (Allis et al., 2007). For example H3 that is tri-methylated at lysine (K) 4 (H3K4me3) is a mark for active genes while H3K9me3 marks heterochromatin linked to inactive genes. The role of such histone-modifications and the corresponding enzymes are well studied in developmental processes but have more recently also be linked to cognitive function.

## Histone-acetylation in memory consolidation

Histone-acetylation has been linked to learning process in rodents already in 1979 when it was found that memory training alters the incorporation of 14C-acetate to histones in different brain regions (Schmitt and Matthies, 1979). Mechanistic proof that histone-acetylation is required for proper memory function was then later demonstrated by the fact that mice which lack HAT activity show impaired memory function (Alarcon et al., 2004; Korzus et al., 2004; Wood et al., 2006; Oliveira et al., 2007; Vecsey et al., 2007; Maurice et al., 2008; Chen et al., 2010; Barrett et al., 2011; McNulty et al., 2012). The very first studies investigated the role of cAMP response element binding protein (CBP) and found that mice which express a mutant form of CBP that lacks the histone-transferase activity (Korzus et al., 2004) or lack the complete CBP gene (Alarcon et al., 2004) show impaired memory formation. This phenotype can be rescued by the administration of HDAC inhibitors (Alarcon et al., 2004). At least 18 HATs are known in mammals and they are subdivided into the GNAT (**G**cn5 ***N***-**a**cetyl**t**ransferases) family, the MYST (**M**OZ, **Y**bf2/Sas3, **S**as2, **T**IP60) family, the p300/CBP family and several other HATs do not belong to any of these families (Allis et al., 2007; Lee and Workman, 2007). Since HATs also acetylate other proteins than histones, a new nomenclature has been suggested and HATs are now referred to as K-lysine acetyltransferases (KATs) followed by a specific number. A substantial amount of data documents a role for KAT3A/CBP in memory function (Alarcon et al., 2004; Korzus et al., 2004; Wood et al., 2006; Vecsey et al., 2007; Chen et al., 2010; McNulty et al., 2012). Moreover, a role for KAT2B/PCAF (Maurice et al., 2008) and KAT3B/P300 in memory function has been suggested. However, the role of the other HATs remains still unknown.

In line with the unanimous findings that impairing HAT function impairs memory formation, it was found that administration of HDAC inhibitors to rodents improves memory consolidation (Levenson et al., 2004; Fischer et al., 2007; Vecsey et al., 2007). Data derived from knock-out mice were able to link such effects to particular HDAC proteins. As such mice that lack neuronal HDAC2 or HDAC3 show better learning than the corresponding wild type littermates (Guan et al., 2009; McQuown et al., 2011). Mice that lack neuronal HDAC1 from early developmental stages do not show altered learning ability in the fear conditioning and water maze paradigms (Guan et al., 2009). However, HDAC1 was found to be essential for fear extinction, a learning process that is critical for the treatment of emotional diseases such as post-traumatic stress disorder (Bahari-Javan et al., 2012). As such, increasing HDAC1 levels in the hippocampus of mice improved fear extinction (Bahari-Javan et al., 2012). Interestingly, the opposite is true for HDAC2 since reducing HDAC2 functions was found to facilitate fear extinction learning in mice (Morris et al., 2013). As for the class II HDACs, deletion of HDAC4 was found to improve memory function in *C. elegans* (Wang et al., 2011). In contrast, mice that lack HDAC4 show impaired memory function and synaptic plasticity (Kim et al., 2012; Sando et al., 2012). Moreover, reduced HDAC4 levels have been linked to mental retardation in humans (Williams et al., 2010). Deletion of HDAC5 in 2-month old mice does not cause memory impairments (Kim et al., 2012). However, aged 10-month old HDAC5 knock-out mice develop mild memory disturbances when compared to age-matched control mice (Agis-Balboa et al., 2013). Mice that lack HDAC6 do not show significant differences in the fear conditioning or water maze paradigm (Govindarajan et al., 2013) and nothing is currently known for the remaining HDACs.

Naturally, the role of HATs and HDACs has been linked to chromatin plasticity and gene-expression. In fact for most of the above described studies, altered memory performance correlated with changes in gene-expression and histone-acetylation. Nevertheless, such findings have to be interpreted with caution since in most cases bulk changes in histone-acetylation were measured and only selected genes were tested for expression or studied via chromatin-immunoprecipitation (ChIP). The mechanistic insight from such data is limited. More recently, researchers have started to study histone-acetylation during memory formation via ChIP-sequencing which allows for a genome-wide analysis. For example, the levels of H4K12 acetylation in the coding region of learning-induced hippocampal genes were linked to better memory performance in young when compared to old mice (Peleg et al., 2010). Another recent study used ChIP-sequencing to study H4K5 acetylation after fear conditioning (Park et al., 2013). Nevertheless, HATs and HDACs likely regulate memory processes also by affecting non-histone proteins, including transcription factors (Lopez-Atalaya et al., 2013). Another issue is that all of the above described studies analyzed brain tissue that contains multiple cell types such as post-mitotic neurons, glia cells and the endothelian cells of the blood vessels. To achieve cell type specificity will be a key issue for future research. While, clearly more research is needed to further understand the role of histone-acetylation in memory function, the current data demonstrate a key role of HATs and HDACs in orchestrating the interplay of synaptic and nuclear plasticity during the encoding of new memories.

## Histone-acetylation in brain in disease

### Alzheimer's disease

Alzheimer's disease is the most common form of dementia in the elderly. On the pathological background of amyloid plaques, neurofibrillary tangles (NFTs) and neuronal cell death patients develop memory disturbances and eventually lose the ability of managing the daily life. A small number of AD cases (ca. 5%) is caused by mutations in amyloid precursor protein (APP) gene or the genes that mediate its processing (familial AD). As a consequence the brain produces more of the AD-associated amyloid-beta 42 (Aß42) peptide (Haass and Selkoe, 2007) that is believed to form toxic oligomers, cause memory disturbances and eventually aggregates into amyloid plaques (Palop and Mucke, 2010; Goate and Hardy, 2012). The majority of the AD cases (95%) are sporadic and characterized by a late onset. Elevated Aß42 levels, amyloid plaques and NFTs are also observed in the sporadic form of AD albeit patients do not carry any of the mutations characteristic for familial AD. It has to be mentioned though that amyloid and NFT pathology does not always correlate with cognitive decline (Snowdon et al., 1997). In fact, while almost all research in the past was focused on amyloid deposition, the corresponding therapeutic clinical approaches failed (Mangialasche et al., 2010). This is certainly also due to the fact that amyloid deposition may begin at least 20 years before the onset of clinical symptoms (Bateman et al., 2012). As such, the tested drugs were probably given to late. However, one should also consider the fact that most of the tested drugs were developed for the 5% familial AD cases but eventually given to sporadic AD patients. Novel therapeutic approaches take this into consideration. For example in an on-going clinical trial an antibody that should lower amyloid pathology is tested in a cohort of familial AD cases (Crenezumab, Genentech, Roche Holding AG). Such approaches are promising but it is not clear if they are also suitable for the 95% of patients that suffer from the sporadic form of AD. Especially since sporadic AD is most likely a result of variable combination of genetic and environmental risk factors it is interesting to consider epigenetic therapeutic approaches in addition, since epigenetic mechanisms including histone-acetylation are key mediators of genome-environment interactions. In fact, to study the role of epigenetics as biomarker and therapeutic targets is a growing field of research.

Taking into account the role of histone-acetylation in memory function and especially the finding that HDAC inhibitors can enhance memory encoding in mice, the potential of HDAC inhibitors for cognitive diseases was soon recognized. In one of the first studies it was found that 4 weeks intra-peritoneal (ip) administration of the HDAC inhibitor sodium butyrate reinstates cognitive function and synaptic plasticity in a mouse model that allowed forebrain-specific and inducible overexpression of the p25 protein (CK-p25 mice) (Fischer et al., 2007). P25 is a subunit of the Cyclin-dependent kinase 5 (CDK5) that is elevated in human AD patients (Patrick et al., 1999) and has been linked to amyloid and tau pathology as well as severe neurodegeneration and memory impairment (Cruz et al., 2003, 2006; Fischer et al., 2005). Importantly, the CK-p25 model is not based on familial AD and sodium butyrate treatment is also effective when administered after the onset of neuronal cell death (Fischer et al., 2007). Subsequent studies confirmed the therapeutic potential of HDAC inhibitors in animal models for amyloid deposition. Different treatment protocols and HDAC inhibitors such as trichostatin A (Francis et al., 2009), phenylbutyrate (Ricobaraza et al., 2009, 2010) valproate (Depakote) or suberoylanilide hydroxamic acid (SAHA, Vorinostat) were used (Kilgore et al., 2010). One study was able to demonstrate that prolonged oral administration of sodium butyrate reinstated memory function in an amyloid-based AD model even when administered at a stage of severe pathology (Govindarajan et al., 2011). While all of these studies showed enhanced memory function in AD mouse models for amyloid deposition, it is interesting to note that none of them reported altered amyloid deposition. A recent study employed an experimental setting in which APPPS1-21 mice orally received the HDAC inhibitor MS-275 (Entinostat) and report reduced neuroinflammation and amyloid plaque load (Zhang and Schluesener, 2013). Memory function was however not tested in this study. Reduced amyloid pathology is also seen in 3xTG AD mice—that develop amyloid and Tau-pathology—that received a novel class II HDAC inhibitor (Sung et al., 2013).

While such studies are encouraging, most of them tested the therapeutic effect of targeting HDACs in models for familial AD. Aging is the most significant risk factor for sporadic AD. It is thus important to note that HDAC inhibitors can reinstate memory function in mice that suffer from age-associated memory loss. For example it was found that SAHA reinstates memory function in old mice. This is mechanistically linked to increased H4K12 acetylation along the coding regions of learning-induced genes (Peleg et al., 2010). Similarly, memory improvement is observed in aged rats treated with sodium butyrate (Reolon et al., 2011) and in senescence-accelerated prone (SAMP8) mice—a model for accelerated aging (Fontán-Lozano et al., 2008).

An important issue is that all of the HDAC inhibitors used so far affect multiple HDAC proteins. To understand which of the 11 HDAC proteins would be the most suitable drug target and which not is therefore of utmost importance. The most conclusive approach to address this issue would be to cross mice that lack a specific HDAC protein to mouse models for AD. Such data is at present only available for the class II HDACs, HDAC5 and HDAC6. When mice lacking HDAC5 are crossed to APPPS1-21 mice, memory function is even further impaired (Agis-Balboa et al., 2013). Mice that lack HDAC6 do not show any obvious phenotype. Crossing HDAC6 knock-out mice with APPPS1-21 mice restores memory function without lowering amyloid deposition (Govindarajan et al., 2013). Reducing HDAC6 levels is mechanistically linked to tubulin-acetylation and improved mitochondria transport (Govindarajan et al., 2013). Moreover, reducing HDAC2 in a mouse model for amyloid deposition via RNA interference also improves memory function and synaptic plasticity (Gräff et al., 2012). Thus, at present it appears that HDAC2 and HDAC6 are the most suitable drug targets to treat memory impairment in AD, while inhibition of HDAC5 should be avoided. Taking into account that deletion of HDAC4 impairs memory function in mice (Kim et al., 2012; Sando et al., 2012), inhibition of this HDAC should also be avoided.

Since HDAC inhibitors improve memory function in AD mouse models, HAT activators should have similar effects. Indeed, recent data shows that administration of HAT agonists improve memory function in wild type mice (Selvi et al., 2010; Chatterjee et al., 2013; Schneider et al., 2013) but data for disease models are not yet available.

In addition to the question which HDAC or HAT would be the best drug target, an important issue to solve is if such a therapeutic approach is disease-modifying or not. Clearly more research is needed to clarify this issue and to elucidate if changes in histone-acetylation are cause or consequence in AD.

**Figure 1:**
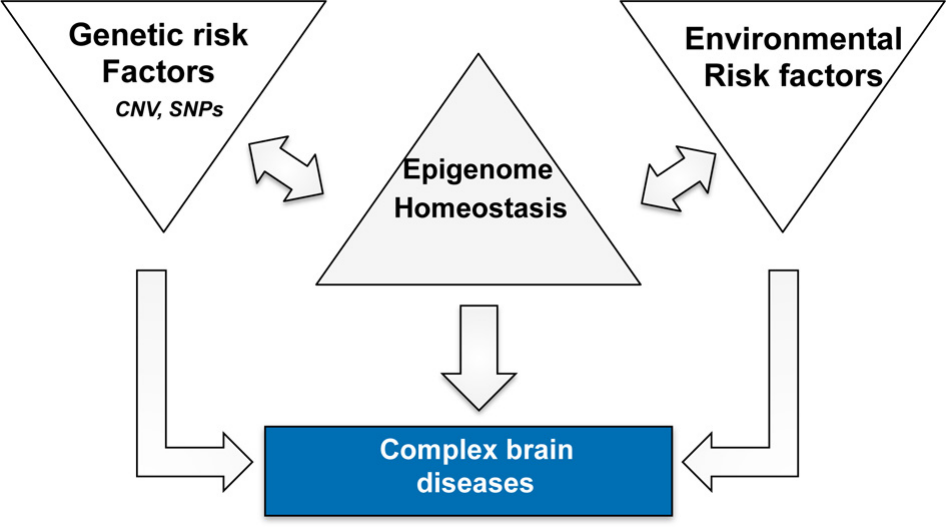
**Complex neuropsychiatric diseases are caused by variable combination of genetic and environmental risk factors that vary on an individual basis and for a given disease**. Epigenetic processes are key regulatory processes that orchestrate genome-environment interactions and there is a substantial amount of literature demonstrating a role for epigenetics in numerous brain diseases. Thus, we speculate that disease pathogenesis is accompanied by a specific epigenetic signature that contributes to the deregulation of cellular homeostasis. The epigenome therefore acts as a bottleneck and provides a bona fide drug target since it may act disease modifying without targeting—or even knowing—all upstream disease causing factors. The signatures are likely to differ for different diseases in that they reflect specific de-regulation of the corresponding enzymatic machinery. One example is the finding that HDAC1 and HDAC2 appear to regulate distinct cellular mechanisms and thus play different roles in the pathogenesis of PTSD and AD.

In this context it is interesting to note that targeting histone-acetylation has not only been linked to AD but also more generally to brain diseases associated with cognitive dysfunction such as drug addiction or schizophrenia (Kumar et al., 2005; Kim et al., 2010; Host et al., 2011; Jordi et al., 2013). We have previously hypothesized that epigenetic processes such as histone-modifications integrate the various environmental stimuli on the level of altered gene-expression and thus provide an important mechanistic link between neuronal activity and adaptive changes in gene-expression that are critical for memory consolidation (Sananbenesi and Fischer, 2009). In this scenario memory consolidation initiates a learning related epigenetic change in the relevant brain cells. In turn, the variable combinations of genetic and environmental risk factors for AD drive a disease-specific epigenetic signature that contributes to pathogenesis. Therefore, an epigenetic therapeutic approach might be beneficial since it would act at the bottleneck where risk factors integrate and drive pathology (Figure 1). The environmental and genetic risk factors for different brain diseases overlap to some extent and we speculate that the different brain diseases reflect endpoints of epigenetic states. This would also imply that the epigenome provides is a link between apparently different brain diseases and we will discuss this idea for AD and post-traumatic stress disorder (PTSD).

## Histone-acetylation in post-traumatic stress disorder. A link to AD?

Emotional disorders often involve excessive fear and anxiety and therapeutic approaches involve inhibition of fear through cognitive behavioral therapy. For example patients that suffer from phobia such as fear of heights would be repeatedly exposed to the frightening stimuli and thereby experiencing habituation and learn that the frightening stimulus is harmless (Deacon and Abramowitz, 2004; Ressler et al., 2004; Fischer and Tsai, 2008).

Similar therapeutic strategies are used to treat patients with posttraumatic stress disorder (PTSD). PTSD can develop as a result of a terrifying and traumatic event, which could involve physical harm or was experienced as extremely threatening (Fischer and Tsai, 2008). Patients suffering from PTSD display extreme traumatic stress to an extent in which normal psychological defense mechanisms fail. Symptoms may involve persistent re-experience of the traumatic event, impairment of social interaction and aversive behaviors such as self-injury. When patients are repeatedly confronted with their feared memories and at the same time experience a feeling of safety, over time this procedure can lead to reduced anxiety and aversive behavior associated with the fear memory (Adshead, 2000; Bisson and Andrew, 2007; Fischer and Tsai, 2008). This process is called fear extinction and substantial progress has been made to understand the underlying molecular mechanisms (Sotres-Bayon et al., 2006; Myers and Davis, 2007; Sananbenesi et al., 2007; Fischer and Tsai, 2008). For the context of this review article it is interesting to note that recently histone-acetylation has been linked to fear extinction. Thus it was found that successful fear extinction correlates with increased H4 acetylation of the BDNF promoter in the prefrontal cortex of mice, which results in enhanced BDNF expression. Moreover administration of the pan-HDAC inhibitor valproate facilitates fear extinction (Bredy et al., 2007; Bredy and Barad, 2008; Heinrichs et al., 2013). In line with such observations i.p. injection of sodium butyrate and intrahippocampal injections of trichostatin A facilitates fear extinction in mice (Lattal et al., 2007). Moreover, administration of SAHA facilitates fear extinction in naive rats (Fujita et al., 2012) and also in rats that had been exposed to the single prolonged stress paradigm, a model for PTSD (Ponomarev et al., 2010; Matsumoto et al., 2013). In contrast to the commonly used C57BL6J mouse it was demonstrated that 129S1/SvlmJ mice are unable to extinct fear memories. However, administration of the HDAC inhibitor valporates promoted extinction in 129S1/SvlmJ (Whittle et al., 2013). Thus, there is substantial data suggesting that HDAC inhibitor treatment facilitates fear extinction and may provide a novel therapeutic avenue to treat PTSD (Maddox et al., 2013). The role of the HATs and HDACs that orchestrate fear extinction learning remains to be elucidated but it was found that mice which lack HDAC2 display enhanced extinction learning of cued fear and conditioned taste aversion memories (Morris et al., 2013).

This data suggest that—similar to its role in memory consolidation—HDAC2 improves extinction learning. In contrast HDAC1 was found to be essential for fear extinction. Thus virus-mediated neuronal overexpression of HDAC1 in the dorsal hippocampus of adult mice significantly facilitated the extinction of contextual fear memories but did not alter other cognitive abilities such as spatial memory, associative memory and working memory (Bahari-Javan et al., 2012).

Successful extinction of fear memories required HDAC1 mediated repression of immediate early gene such as *c-fos* and *egr-2* that was achieved via decreased Histone H3 Lysine 9 (H3K9) acetylation, a marker for transcriptional activation (Kouzarides, 2007) and subsequent H3K9 trimethylation (H3K9me3), a marker for heterochromatin. In line with this data knock down of HDAC1 prevented transcriptional repression of *c-fos* and *egr-2* resulting in impaired fear extinction. This data suggests that histone-acetylation is a critical regulatory process of fear extinction. However it will be critical to elucidate the role of the relevant enzymes and define the histone-acetylation patters for the relevant genes. Especially since HDAC1 and HDAC2 appear to have opposing roles in fear extinction. This is however also true for AD, where HDAC2 but not HDAC1 inhibition is able to reinstate memory function (Guan et al., 2009; Gräff et al., 2012).

In conclusion there is substantial evidence that the mechanisms that orchestrate histone-acetylation are involved in fear extinction and there is indeed further data suggesting a role of epigenetic mechanisms in the pathogenesis of PTSD (Zovkic et al., 2013). Especially risk factors such as early life stress have been linked to epigenetic changes that predispose an individual to the development of PTSD (Domschke, 2012) and epigenetic changes have been observed in PTSD patients (Uddin et al., 2010; Lee et al., 2011; Rusiecki et al., 2012). Interestingly, individuals that suffer from PTSD at young age, have almost double the risk to develop AD at old age (Yaffe et al., 2010; Burri et al., 2013). On the basis of such data, we hypothesize that the pathogenesis of PTSD correlates with epigenetic changes that contribute to disease phenotypes. Albeit this would need to be studied in greater detail, there is substantial evidence that the epigenome undergoes further changes during aging which contribute to memory decline. Thus, the epigenome of an individual that suffered from PTSD would be more susceptible to further age-related changes—or other risk factors for AD—which increases the risk to eventually develop dementia (Figure 1).

## Conclusion

The epigenome transfers environmental stimuli into long term adaptive changes and thus provides to neurons a mechanisms that allows for the compromise between stability and flexibility. Epigenetic changes have been observed in various brain diseases and epigenetic drugs such as HDAC inhibitors show beneficial effects in animal models for apparently different brain diseases such as AD or PTSD. Interestingly, epidemiological data links early life psychiatric diseases such as PTSD to late onset AD. One common feature amongst such diseases is that they are associated with epigenetic alterations and it can be speculated that disease associated epigenetic changes—if not treated—predispose the system for further de-regulation in response to additional environmental risk factors. Thus, the various brain diseases may be viewed as different places in the epigenetic landscape that are off the main path but still interlinked. Epigenetic changes can be transient, long-lasting or even transmitted across generations but they are always reversible and thus hold great potential for future therapeutic approaches that aim to treat the correct patient population for a given disease.

### Conflict of interest statement

The authors declare that the research was conducted in the absence of any commercial or financial relationships that could be construed as a potential conflict of interest.
